# Executive functions in schizophrenia

**DOI:** 10.4103/0019-5545.46069

**Published:** 2005

**Authors:** S. Sabhesan, S. Parthasarathy

**Affiliations:** * Reader in Psychiatry Thoothukudi Medical College, Thoothukudi; ** Consultant Psychiatrist, Madurai

**Keywords:** Executive functions, neurocognition, schizophrenia

## Abstract

**Background:**

Executive functions constitute the core deficit in schizophrenic illness and have been related to structural and functional deficits, cognitive impairments and final outcome.

**Aim:**

To study the various dimensions of executive functions such as goal formulation, planning, behavioural programming and effective performance.

**Methods:**

By using direct and indirect clinical neuropsychological methods, 31 patients were studied neuropsychologically by the trail-making test (TMT), Raven matrices and fluency tests, and their symptom patterns were quantified using the Positive and Negative Syndrome Scale (PANSS).

**Results:**

The patients had varying degrees of involvement of different dimensions of executive functions. There was an inverse relationship to TMT and a positive correlation with Raven matrices and fluency tests.

**Conclusion:**

The dimensions of executive functions did not show any significant relationship with age, duration of illness or most scores in PANSS. Our findings are relevant for remediation and rehabilitation measures.

## INTRODUCTION

Executive functions include the capacity to formulate goals, plan and organize goal-directed behaviour, carry out goal-directed behaviour fully and effectively, and monitor and self-correct one's behaviour as needed.^1^ Luria referred to the loss of meaningful, directed behaviour resulting from frontal lobe lesions, in the absence of marked disturbances in motor activity or sensitivity, gnosis or praxis as a construct in rehabilitation.^2^ Disturbances in executive functions have been structurally related to the prefrontal lobes, neuropsychologically to defective performance in tests of problem-solving abilities, and functionally to disability in maintaining a complex behaviour sequence without help.^3^,^4^

Schizophrenics have varying grades of impairment of executive processes, which result in difficulties during extended and multifaceted interpersonal interactions. Carter and Barch relate the neural basis of executive functions to the dorsolateral prefrontal cortex and anterior cingulate cortex, and show that functional imaging studies identified disturbances in other areas as well, particularly the temporolimbic region.^5^ Disturbances in higher cognitive functions may have an engaging relationship to the functional hypofrontality observed among these patients during positron emission tomography (PET) and functional MRI studies. Subtle cognitive deficits have been described in working memory, information processing, supervisory attentional system and others.^6^,^7^ Weickert and Goldberg suggest that deficits in executive and attentional functions constitute a necessary and sufficient type of cognitive impairment in schizophrenia.^8^ Deficits in non-executive domains of neurocognition are relevant in understanding schizophrenia and an interactional or attributory model may relate the multiple functional impairments to explain the heterogeneity of clinical presentation and variations in functional outcome.^9^

Evaluation of the component elements of executive functions has been attempted with neuropsychological tests such as card sorting tests, maze tests, tower tests, tinkertoy tests and fluency tests, and with specific test batteries such as the Behavioral Assessment of Dysexecutive Syndrome.^10^,^11^ However, a conceptual paradox arises in attempting to study executive functions through standard neuropsychological measures because most testing situations are standardized and deficits in initiation and goal-directed behaviour might not be apparent.^9^,^10^ Objective and quantitative non-psychometric behavioural assessment in clinical neuropsychology is relatively new and comprises direct and indirect methods.^12^ Direct methods include observation of the individual in the native setting or in an analogue situation, and indirect methods involve interviewing, rating by others and self-reports. Such clinical neuropsychological evaluation would be simple and reliable, and useful in planning rehabilitation.

The present study aims to use a schedule for clinical neuropsychological evaluation of executive functions and to know its correlation with other cognitive impairments and clinical symptomatology.

## METHODS

The study was conducted in the private clinic of the senior author (SS) between May 2002 and October 2002. Patients with the following criteria were chosen for the study:

- those between 20 and 50 years of age
- those regularly followed up for at least 6 months
- those meeting the ICD-10 criteria for schizophrenic illness^13^
- those without co-morbid conditions such as drug abuse, affective symptoms or other neuropsychiatric problems
- those consenting to cooperate during the neuropsychological assessment, though motivation levels were varied
- those in whom electroconvulsive therapy (ECT) had not been administered during the previous 6 months.

A total of 31 patients were selected and were interviewed along with their caretakers. Drugs were stopped for a day before the day of examination. The patients were at varying levels of recovery. The following tools were used:

1. ICD-10^13^
2. Executive Functions Assessment Schedule (*see* p. 26)
3. Trail-making test (TMT)^14^
4. Raven matrices^15^
5. Fluency tests^10^
6. Positive and Negative Syndrome Scale (PANSS)^16^

Those who fulfilled the criteria were selected by the senior author and each patient' behavioural repertoire was collected from the relatives. The tests (TMT, Raven matrices, fluency and PANSS) were administered by the junior author (SP). At the end of the testing, the behaviour during testing was discussed and the senior author scored the Executive Functions Assessment Schedule.

The sociodemographic details reflected the random choice of the relatively homogeneous population. Of the 31 patients, 23 were men (74%). Fifteen patients (48%;) were between 20 and 29 years of age, 14 (45%) between 30 and 39 years and the rest (7%) between 40 and 50 years. Fourteen patients (45%) were from rural areas and the rest from urban. Five were illiterate (16%), 10 (32%) had primary school education, 9 (29%) had 5–10 years of education and the remaining 7 had more than 10 years of education. Four patients had been ill for less than 2 years, 8 patients (26%) for 2–5 years and the rest (61%) for more than 5 years. Six patients (19%) had been on treatment for less than 2 years, 11 (36%) for 2–5 years and 14 (45%) for more than 5 years.

Patients were interviewed in detail along with the relatives and were requested to do the neuropsychological tests, after instructions were given. Details from the relatives, narration of the activities of daily living and their behaviour during unfamiliar and non-habitual tasks were enquired into. Their behaviour during the psychological testing was observed and the mechanism of their operations discussed at the end of the testing. Based on the totality of these findings, the dimensions of executive functions were scored. Neuropsychological tests were scored routinely.

As per the explorative design of the study and nature of the tools, simple correlation techniques were used to analyse the data.

## RESULTS

Clinical interviews of the patients enabled the quantitation of components of positive and negative syndromes as per PANSS (Table 1). Central tendencies and range of dispersion were calculated for all the scores. The correlation matrix of the dimensions of executive functions showed that each was correlated to every other dimension and to the total, at statistically significant levels (Table 2).

**Table 1:** Details of scores in neuropsychological assessment and PANSS (*n*=31)

	Mean	SD	Maximum	Minimum
Executive functions				
I	2.44	0.5	3	2
II	2.13	0.7	3	1
III	0.75	0.7	2	0
IV	0.44	0.6	2	0
Total	5.75	2.1	10	3
Trail-making test				
I	3.47	2.9	10	1
II	14.91	8.3	45	5
Raven test				
Score	19.69	11.74	45	6
Time (minutes)	20.68	13.50	60	11
Fluency				
Linguistic				
I	8.09	3.0	19	2
II	4.88	1.8	8	2
Category				
I	5.38	3.5	13	0
II	4.09	2.7	8	0
PANSS				
Positive syndrome	15.78	6.3	29	7
Negative syndrome	21.84	9.6	42	7
General psychopathology	34.19	10.6	59	19
Anergia	10.72	4.3	18	4
Thought disturbance	9.81	3.8	18	4
Activation	5.16	1.8	8	3
Belligerence	5.97	2.4	11	3
Depression	9.09	3.9	20	5

PANSS: Positive and Negative Syndrome Scale

**Table 2:** Intercorrelations among executive functions†

	I	II	III	IV	Total
I		0.55*	0.62*	0.59*	0.79*
II			0.61*	0.59*	0.83*
III				0.74*	0.89*
IV					0.86*
Total					

† Components I-IV as defined in Executive Functions Assessment Schedule (see page 26)

* p<0.01

Degrees of freedom (df) = 29

Associations between executive functions and neuro-psychological test performance showed that performance in TMT I and II was inversely correlated and that most of the correlations were statistically significant. Raw scores in Raven matrices showed a positive correlation with all dimensions of executive functions. The time taken for completion of the test correlated significantly with goal formulation and effective performance. Linguistic fluency and category fluency were both positively correlated with all dimensions of executive functions and most of the associations were statistically significant. Age and duration of illness were not significantly correlated though both showed a consistently inverse relationship. Scores in positive and negative syndromes did not show significant correlation, though negative syndrome, general psychopathology and anergia were consistently negative in their associations. Except for a significant correlation between belligerence and behaviour programming, there was no statistically significant association in all the other attributes (Table 3).

**Table 3:** Correlations of executive functions with age, duration of illness, neuropsychological performance and PANSS

	I	II	III	IV	Total
Age	−0.29	−0.12	−0.27	−0.19	−0.25
Duration of illness	−0.26	−0.22	−0.24	−0.01	−0.21
Trail-making test					
I	−0.51***	−0.24	−0.59***	−0.47***	−0.53***
II	−0.44**	−0.39*	−0.41*	−0.46***	−0.50***
Raven test					
Score	0.58***	0.49***	0.54***	0.74***	0.69***
Time	0.38*	−0.04	0.21	0.37*	0.25
Fluency					
Linguistic					
I	0.40*	0.33	0.62***	0.44**	0.53***
II	0.15	0.51***	0.42**	0.21	0.40*
Category					
I	0.47***	0.53***	0.65***	0.55***	0.66***
II	0.31	0.46***	0.68***	0.51***	0.59***
PANSS					
Positive symptoms	−0.18	0.16	0.26	0.04	0.11
Negative symptoms	−0.00	−0.01	−0.06	−0.09	−0.05
General psychopathology	−0.22	−0.14	0.10	−0.30	−0.15
Anergia	−0.07	−0.05	−0.03	−0.20	−0.15
Thought disorder	−0.15	0.17	0.19	−0.05	0.07
Activation	0	−0.05	0.33	−0.06	0.07
Belligerence	−0.22	0.02	0.36*	0.14	0.11
Depression	−0.20	−0.15	0.07	−0.29	−0.16

* p<0.05;

** p<0.02;

*** p<0.01

Degrees of freedom (df) = 29

PANSS: Positive and Negative Syndrome Scale

## DISCUSSION

Most studies on executive functions have used flexible neuropsychological tests such as Wisconsin card sorting test, tinkertoy test and tower tests. However, the motivational component might not be measurable under normal test situations and executive functions cannot be tested in the absence of reliable quantitation of motivation. Moreover, motivation and spontaneity studied in a formal one-to-one situation during psychological testing might not reflect the situation in the natural setting. The Executive Functions Assessment Schedule used in the present study addressed these difficulties by formulating a four-dimensional module, studied by both direct and indirect methods of behavioural quantitation. The study of executive functions in both the natural and the unfamiliar setting of psychometric testing enabled a better assessment of the individual.

The dimensions were studied individually. Goal formulation was assessed by identifying the motivational levels, awareness of environmental situations and the ability to formulate goals, and scored along hierarchy of functions. Thus, increasingly complex situations were quantified as being related to basic visceral-related functions, to habitually orchestrated behaviour or to innovative formulation to meet unfamiliar situations. Similar scoring on the basis of application of experiences and ability to abstract was used to quantify planning. Behavioural programming was scored on the ability to perform unstructured tasks. Effective performance was quantified on the basis of the subject's ability to identify defects in behaviour, plan corrective measures and apply the correction in both structured and unstructured tasks.^10^,^12^,^17^

The dimensions of executive functions showed a significant degree of positive correlation among themselves and with the total score. The schedule had a high degree of internal consistency. The extent of association indicated that the subfunctions were related to each other either structurally through a shared anatomical basis, or functionally by being a part of an integrated physiological circuitry, subserving an essential component of behavioural execution.

Scores in executive functions showed that standard deviations increased in effective performance where the variance was wide. Mean scores were relatively high in goal formulation (81%) and low in effective performance (22%). Though the present study did not envisage comparison with depressives or neurotics, central tendencies and dispersion showed that individual schizophrenics varied considerably and that different dimensions were affected to varying degrees in individuals. Carter and Barch related the disturbances to impaired context presentation and maintenance, impaired attention and impaired performance monitoring, and surmised that various executive defects exist, with each contributing to a different cognitive ability.^5^

Correlation of executive functions to demographic, clinical and neuropsychological data was carried out. Age and duration of illness had a consistently inverse, but statistically non-significant, relationship. Thus, defects in executive functions were not related to increasing age or length of illness and were independent facets of schizophrenic illness.

The TMT has been used as a test of complex visual scanning, involving motor speed and mental tracking. It gave a good idea of how effectively the patient responded to a complex visual array, followed a sequence mentally, dealt with more than one stimulus at a time, and was flexible in shifting the course of ongoing activity.^10^ Impaired selection of task-relevant information and inability to discriminate signal from noise characterized the attentional deficits in schizophrenics.^9^ Patients in the present study showed a uniform, negative and significant correlation among all dimensions of executive functions and both TMT tests (except planning and TMT I), demonstrating that executive functions were associated with an intact vigilance mechanism.^4^

Raven matrices required the subjects to conceptualize spatial, design and numerical relationships ranging from the obvious to the abstract. Error tendencies were observed and questioned at the end to reveal the patterns of error in concept formation.^10^ Poor raw scores (mean 19.69±11.7) reflected the general intellectual decline among schizophrenics.^8^ Raw scores had a significant and positive correlation with all dimensions of executive functions. Time taken for completion of the test showed a varying relationship to executive functions. Intact goal formulation and effective performance were associated with a longer duration. Observation of error tendencies and error patterns showed that patients with impaired functions gave random answers, thus reducing the total time needed for completion.

Fluency tests involved active search and monitoring and included word generation according to an initial letter or category. Category fluency provided a better structure and vocabulary involved accessing stored knowledge.^6^ Executive functions had a positive correlation with both linguistic and category fluency, more so with the latter. Carter and Barch found that schizophrenics exhibited deficits in both production and comprehension of language, and that poor executive functions were causally related to linguistic impairments.^5^

The psychopathology in schizophrenics reflected the tertiary outcome of innate cognitive changes. The PANSS measurement was derived from behavioural information and a 7-point rating of 30 symptoms.^16^ Most investigators agree that a positive syndrome has no significant relationship with any dimension of executive functions.^9^ Compared to positive symptoms, negative symptoms were more stable, persistent and predictive and, in the present study, had a consistent negative relationship with executive functions, though not at statistically significant levels. Similar consistent and non-significant inverse relationships were noted with scores in general psychopathology and anergia.

Composite measures of neurocognition explained 20%–60% of the variance in functional outcome and were necessary for optimal adaptation of schizophrenic patients. Functional neuroimaging studies have shown that higher cognitive deficits were related to physiological dysfunction in circuits underlying executive functions in the brain.^5^ Though executive functions, cognition and social outcome vary widely in their subfunctions, the relationship between specific dimensions of executive functions and specific aspects of cognitive and social outcome need to be understood for planning potential pharmacological and rehabilitative interventions. Pencil and paper exercises to improve executive functions have been formulated to enhance cognition and reduce clinical symptoms and, if generalizable, would help in future management.^18^ For all pharmacological and non-pharmacological cognitive remediation, executive functions constitute the focus for further exploration.

**Table:** 

EXECUTIVE FUNCTIONS ASSESSMENT SCHEDULE	
Dimensions of executive functions included in the schedule are goal formulation, planning, behavioural programming and effective performance. Details of day-to-day activities, anecdotal details narrated by the relatives, observations during the interview and observations of the technique and behaviour during performance of neuropsychological tests were considered. The tasks were graded as related to visceral functions (such as eating, dressing), other habitual and routine activities, or unfamiliar and non-habitual functions requiring innovative solutions. Scoring in each dimension was as follows:
**I Goal formulation:** The patient's level of motivation (spontaneous or induced), awareness of the context (relation of goal to context) and ability to formulate appropriate goals were considered in scoring this dimension:	
Nil	0
Visceral-related functions	1
Habitual activities	2
Unfamiliar acts	3
**II Planning:** Ability to plan depends on one's applying past experiences to the present task and the ability to abstract the present problem. Scoring was as follows:	
Nil	0
Visceral-related functions	1
Habitual activities	2
Unfamiliar acts	3
**III Behavioural programming:** Ability to programme activities was scored on whether the patient was able to carry out plans to known and practised acts or to unfamiliar and non-habitual tasks.	
Nil	0
Structured tasks	1
Unstructured tasks	2
**IV Effective performance:** Self-monitoring consists of the ability to identify defects, plan corrective measures and apply the correction properly. Scoring depended upon the ability to feedback correction of habitual acts or even unfamiliar acts.	
Nil	0
Structured tasks	1
Unstructured tasks	2
**Maximum total score:** 10	
